# Coated Zinc Oxide Improves Growth Performance of Weaned Piglets *via* Gut Microbiota

**DOI:** 10.3389/fnut.2022.819722

**Published:** 2022-02-25

**Authors:** Yiwei Sun, Ning Ma, Zengkai Qi, Meng Han, Xi Ma

**Affiliations:** State Key Laboratory of Animal Nutrition, College of Animal Science and Technology, China Agricultural University, Beijing, China

**Keywords:** coated zinc oxide, post-weaned diarrhea, gut microbiota, intestinal health, intestinal barrier

## Abstract

Weaned piglets stayed in transitional stages of internal organ development and external environment change. The dual stresses commonly caused intestinal disorders followed by damaged growth performance and severe diarrhea. High dose of zinc oxide could improve production efficiency and alleviate disease status whereas caused serious environmental pollution. This research investigated if coated ZnO (C_ZnO) in low dose could replace the traditional dose of ZnO to improve the growth performance, intestinal function, and gut microbiota structures in the weaned piglets. A total of 126 cross-bred piglets (7.0 ± 0.5 kg body weight) were randomly allocated into three groups and fed a basal diet or a basal diet supplemented with ZnO (2,000 mg Zn/kg) or C_ZnO (500 mg Zn/kg), respectively. The test lasted for 6 weeks. C_ZnO improved average daily gain (ADG) and feed efficiency, alleviated diarrhea, decreased the lactulose/mannitol ratio (L/M) in the urine, increased the ileal villus height, and upregulated the expression of Occludin in the ileal tissue and the effect was even better than a high concentration of ZnO. Importantly, C_ZnO also regulated the intestinal flora, enriching *Streptococcus* and *Lactobacillus* and removing *Bacillus* and intestinal disease-associated pathogens, including *Clostridium_sensu_stricto_1* and *Cronobacter* in the ileal lumen. Although, colonic microbiota remained relatively stable, the marked rise of *Blautia*, a potential probiotic related to body health, could still be found. In addition, C_ZnO also led to a significant increase of acetate and propionate in both foregut and hindgut. Collectively, a low concentration of C_ZnO could effectively promote growth performance and reduce diarrhea through improving small intestinal morphology and permeability, enhancing the barrier function, adjusting the structure of gut microbiota, and raising the concentration of short-chain fatty acids (SCFAs) in the weaned piglets.

## Introduction

These years, early weaning techniques were commonly used in factories with the acceleration of the intensive breeding process, which directly caused severe diarrhea in piglets under multiple stresses, such as shifts in nutrition and environment (1, 2). ZnO had been recognized as a diarrhea inhibitor and growth promoter in a high dose, whereas ZnO was mostly consumed into zinc ion in the acidic environment of the gut and then eliminated from the body (3). Compared with inorganic zinc ions, ZnO reached better growth performance and was less toxic which meant the main form that worked was a molecular form (4).

The coating was a common means of drug delivery, which could improve chemical stability and bioavailability. Given that 75–90% of supplemental ZnO in the diet could not be absorbed resulting in severe environmental pollution (5), ZnO had been limited to 1,600 mg Zn/kg in the diet of weaned piglets these years. However, the traditional volume of ZnO was at least 2,000 mg Zn/kg in the previous researches to reach the effect of improved mucosal barrier function and reduced diarrhea incidence (6). Thus, encapsulation seemed like an effective method to solve the problem that a lower dose of ZnO could not function as before. With the protection of the covering layer, the ZnO in the inner core could be retained to reach the intestine, where ZnO performed its primary function. Some studies had indicated that ZnO (at least 380 mg Zn/kg) supported on carriers, such as smectite, or coated with enteric materials, such as lipid, reached the same effect of ZnO in high dose (3, 7), while lower content of ZnO (100 or 200 mg Zn/kg) did not make any difference on growth performance and stool consistency (8, 9). The observation concluded that coating treatment was an effective means to reduce the use of ZnO to some extent. Thus, based on the effective delivery of lipid-coated products (8), our research adopted a lipid-encapsulated method to protect ZnO from the acidic environment in the gut. After efficiently reaching the intestine, coated ZnO (C_ZnO) released the ZnO particles under the effect of lipase. However, the function of lipid C_ZnO composite on the diarrhea rate, intestinal barrier proteins, and inflammatory status remained controversial in the weaned piglets (8, 10).

Postweaned diarrhea was commonly companied with disturbed gut microbiota (11, 12). ZnO and C_ZnO could both act as microecological regulators. ZnO treatment could decrease the abundance of the opportunistic pathogen, Campylobacterales, accompanied by the increase of Enterobacteriales in the ileum, while *Methanobrevibacter* dramatically gathered in the colon (13). However, lipid C_ZnO-induced changes of gut microbiota were less known. Given that intestinal flora was crucial for host health and had been proved as a target for disease treatment (14–16), regulating intestinal microbial structure might be an important aspect to realize its function. However, the study of C_ZnO in the microbiological regulation of whole intestine seemed relatively deficient at present. Thus, we would focus on this aspect to investigate the effects of C_ZnO in this article.

## Materials and Methods

### Animals and Treatments

A total of 126 crossbred piglets (Duroc × Landrace × Yorkshire, weaned at 21 days) with an initial body weight (IBW) of 7.0 ± 0.5 kg, were allocated into three groups, six replicates per group, and seven pigs per pen (2.0 m × 1.5 m) based on body weights. Piglets were fed with a basal diet (CON) or a basal diet supplemented with ZnO (2,000 mg Zn/kg) and C_ZnO (500 mg Zn/kg), respectively. The feeding experiment lasted for 42 days. The basal diet was formulated according to the National Research Council (NRC 2012) recommendations to meet or exceed the nutritional requirements (Table 1). All feed and water were available *ad libitum*. The ambient temperature was maintained at 26 ± 2°C and relative humidity was controlled at 60 ± 5%.

**Table 1:** The ingredient and nutrient content of the basal diets used in two growth phases of weaned piglets (dry matter basis, %).

**Ingredients**	**Content (%)**		**Analyzed nutrient composition**	**Content (%)**	
	**Phase 1^a^**	**Phase 2**		**Phase 1**	**Phase 2**
Corn	61.00	63.25	DE, MJ/kg^c^	14.79	14.64
Soybean	13.30	15.50	CP, %	17.96	18.10
Puffed full-fat soybeans	12.10	8.00	Lysine, %	1.48	1.38
Fish meal (64.6%)	3.00	4.00	Met, %	0.43	0.41
Whey powder (3.8%)	4.15	4.00	Thr, %	0.89	0.85
Soybean oil	2.78	2.27	Trp, %	0.24	0.24
Dicalcium phosphate	1.20	0.66	Ca, %	0.80	0.70
Limestone	0.85	0.80	TP, %	0.61	0.54
Salt	0.15	0.30	AP, %	0.41	0.33
L-lys	0.60	0.45			
DL-met	0.12	0.08			
Thr	0.21	0.15			
Trp	0.04	0.04			
Premix^b^	0.50	0.50			
Total	100.00	100.00			

a *Phase 1 referred to the weight of piglets ranging from 7 to 11 kg and phase 2 referred to the weight of piglets ranging from 11 to 25 kg*.

b *Premix provided the following per kg of diet: vitamin A, 12,000 IU; vitamin D3, 3,000 IU; vitamin E, 30 IU; vitamin K3, 2.5 mg; vitamin B12,20.0 μg; riboflavin, 4.0 mg; pantothenic acid, 12.5 mg; niacin, 40 mg; choline chloride, 400 mg; folacin, 0.7 mg; thiamine 2.5 mg; pyridoxine 3.0 mg; biotin, 70 μg; Mn, 30 mg; Fe, 100 mg; Zn, 80 mg (ZnO); Cu, 90 mg; I, 0.25 mg; Se, 0.15 mg*.

c *Digestible energy content of the diet was calculated using energy values for the ingredients obtained from NRC*.

*DE, digestible energy; CP, crude protein; Met, methionine; Thr, threonine; Trp, tryptophan; Ca, calcium; TP, total phosphorus; AP, available phosphorus*.

### Growth Performance and Diarrhea Rate

All the piglets were individually weighed at the beginning (day 0) and end (day 42) of the experiment, and average daily gain (ADG) was calculated. The feed intake of each pen was recorded to calculate the average daily feed intake (ADFI) and feed to gain ratio (F/G). The incidence of diarrhea for each pen was observed and recorded at 08:00 and 14:00 h each day during the experimental period. Diarrhea rate was calculated according to the formula: diarrhea rate = Σ (the number of pigs with diarrhea per pen × days of diarrhea) / (total number of piglets × 21 days) × 100% (17).

### Lactulose/Mannitol Test

The lactulose/mannitol (L/M) test was performed as described previously (1). The urine of six piglets that starved 6 h of each group was collected for baseline urinary sugar measurement. Then, the experimental piglets were orally administrated with 5 ml lactulose (0.4 g/ml; Sigma) and mannitol (0.2 g/l; Sigma). And urine was collected over a 6 h starving period from each pig. Concentrations of lactulose and mannitol in urine were determined by an enzymatic spectrophotometric method.

### Sample Collection

In the morning of the 42nd day, six pigs from each group were sacrificed. Intestinal tissue segments of about 2 cm were immediately separated from the same sections of the ileum and colon with carefulness to avoid squeezing, and fixed in 4% paraformaldehyde solution for intestinal morphology analysis. The remaining intestinal segments and digesta samples recovered from the ileum and colon were transported to liquid nitrogen quickly.

### Intestinal Morphology Analysis

All samples were fixed in 4% paraformaldehyde for 48 h and then embedded in paraffin. Sections of 3 μm were cut and stained in H&E. Each sample was set three duplications and the microstructures of the ileum and colon were analyzed by using a microscope (BX51 type, Olympus Corporation, Japan). The villus height and the crypt depth were measured, and the ratio of villus height to crypt depth of each sample was calculated.

### Western Blotting Analysis

Ileal and colonic samples were collected to measure the relative expressions of Claudin-7 and Occludin. Briefly, total proteins were extracted, and then the concentration of protein was determined by a bicinchoninic acid (BCA) kit (Pierce, Rockford, Illinois, USA). Next, the proteins were denatured, subjected to sodium dodecyl sulfate-polyacrylamide gel electrophoresis (SDS–PAGE), and transferred to the polyvinylidene fluoride (PVDF) membranes. The membranes were then blocked with 5% skim milk powder, followed by overnight incubation at 4°C with primary anti-β-tubulin (DSHB, Beijing, China), anti-Claudin-7 (Abcam, Nanjing, Jiangsu, China), and anti-Occludin (Abcam, Nanjing, Jiangsu, China) antibodies and appropriate secondary antibodies (CST, Nanjing, Jiangsu, China) for 2 h at room temperature. Finally, the bounds were visualized by the LI-COR Infrared Imaging System (Odyssey, Lincoln, NE).

### Extraction of Microbial DNA From Intestinal Digesta

Microbial genomic DNA was extracted from ileal and colonic digesta of piglets using a Stool DNA kit (Omega Bio-tek, Norcross, Georgia, USA). Samples were homogenized, purified, and diluted to a final concentration of about 30 ng μl^−1^. Then, the PCR amplification procedure was set as follows: 95°C for 5 min and then 25 cycles at 95°C for 30 s, 56°C for 30 s and 72°C for 40 s, finally 72°C for 10 min. PCR mixture of total 50 μl concluded 30 ng DNA sample, 2 μl of each Primer [338F (5′-ACTCCTACGGGAGGCAGCA-3′) and 806R (5′-GGACTACHVGGGTWTCTAAT-3′)], 4 μl dNTPs (2.5 mM), 5 μl Fast Pfu Buffer and 3 μl Fast Pfu DNA Polymerase. The PCR products were examined on a 2% agarose gel. Then, the purified amplicons were sequenced on Illumina HiSeq 2000 platform according to protocols of Majorbio Bio-Pharm Technology Co., Ltd. (Shanghai, China) to detect the two hypervariable regions of 16S rRNA, V3 and V4 regions. The sequence analysis was performed on QIIME and UPARSE, which resulted in sequences over 50 bp retained for phynotype analysis and clustered with 97% similarity.

### Ion Chromatography

The concentrations of ileal and colonic short-chain fatty acids (SCFAs) were analyzed by ion chromatography. About 0.6 g of sample was resuspended in 10 ml ultrapure water, then the mixture performed ultrasonic treatment for 30 min and centrifuged at 5,000 rpm for 10 min to obtain the supernatant. The supernatant was diluted 20 or 50 times for the foregut and hindgut, individually. After filtering with a 0.20 μm nylon membrane filter, the solution was poured into an ion chromatography system (Dionex ICS-3000).

### Bacterial Isolation, Characterization, and Physiological Identification

The ileal digesta of C_ZnO-treated piglets was diluted and pelleted on the De Man, Rogosa, and Sharpe (MRS) medium for a period of 48 h at 37°C in an anaerobic workstation (Longyue, Shanghai, China) for the isolation of *Streptococcus*. Meanwhile, the *Bacillus* was isolated and cultured on the Luria-Bertani (LB) plate at 37°C in the incubator. Then the single colonies were sequenced using 16S primers: 27F (5′-AGAGTTTGATCMTGGCTCAG-3′) and 1492R (5′-GGTTACCTTGTTACGACTT-3′), followed by comparing with the NCBI sequence database with basic local alignment search tool (BLAST) and constructing a phylogenetic tree by using MEGA version 11 (The Biodesign Institute, Tempe, AZ, USA).

For Gram staining, a drop of bacterial suspension was pelleted on the glass slide and fixed with flame. Then it was stained with crystal violet for 1 min and washed with running water. Iodine staining was applied and incubated for 1 min, followed by washing with running water to remove the dye. After decolorization with alcohol for 30 s and thorough rinse, the microbe was stained with safranine for 1 min and washed with running water. The slide was finally observed *via* microscope (BX51 type, Olympus Corporation, Japan).

The effect of interbacterial inhibition was detected by the oxford cup method. *Bacillus* in the logarithmic phase was diluted to 10^5^ CFU/ml as an indicator. And the antibacterial ability of *Streptococcus* was detected, while the normal saline was used as a negative control.

For detecting the tolerance to C_ZnO and ZnO, bacteria were coincubated with C_ZnO and ZnO in graded concentrations ranging from 0 to 1 g Zn/L for 8 h to reach the stationary phase. The bacteria solution was pelleted on the culture after dilution, then the colonies were counted. The result was calculated according to the formula: tolerance = log_10_CFU (numbers of bacteria in different concentrations of C_ZnO or ZnO culture/numbers of bacteria in normal culture).

### Statistical Analysis

Statistical analysis was carried out using SAS version 9.1 (SAS Institue Inc, Cary, NC, USA). ANOVA followed by Tukey's multiple range tests were performed to assess statistical significance. The results were shown as mean ± SEM. *P* < 0.05 was considered significant, and *P* < 0.01 was strongly significant.

## Results

### C_ZnO Improved the Growth Performance, Diarrhea Rate, and Intestinal Permeability

Coated ZnO improved end bodyweight (EDW), ADG, and F/G compared to the CON group and even the ZnO group (*P* < 0.05). And the ADFI showed no difference among all groups (*P* > 0.05). ZnO and C_ZnO both effectively reduced the diarrhea incidence in the period of the experiment (*P* < 0.05). In addition, ZnO and C_ZnO also significantly decreased the intestinal permeability compared with the CON group (*P* < 0.05) as indicated by the L/M (Table 2).

**Table 2:** Effects of coated ZnO (C_ZnO) on the growth performance of piglets.

**Items**	**Treatment**		
	**CON**	**ZnO**	**C_ZnO**
**Growth performance**			
IBW, kg	7.0 ± 0.50	7.0 ± 0.50	7.0 ± 0.50
EBW, kg	24.30 ± 0.51^c^	25.67 ± 0.54^b^	26.8 ± 0.55^a^
ADG, g	412.10 ± 35.23^c^	444.50 ± 33.76^b^	471.50 ± 29.32^a^
ADFI, g	704.69 ± 54.02	733.43 ± 46.19	749.69 ± 34.46
F/G	1.71 ± 0.25^a^	1.65 ± 0.24^b^	1.59 ± 0.22^c^
**Diarrhea incidence, %**			
1–3 week	31.90 ± 0.15^a^	23.10 ± 0.10^b^	22.10 ± 0.11^b^
3–6 week	25.10 ± 0.09^a^	17.30 ± 0.11^b^	15.50 ± 0.10^b^
L/M	0.599 ± 0.02^a^	0.249 ± 0.07^b^	0.234 ± 0.01^b^

*Data of growth performance and diarrhea incidence were shown as mean ± SEM (n = 42). Data of L/M were shown as mean ± SEM (n = 6). Different superscript within a row means significantly different (P <0.05)*.

*IBW, initial body weight; EBW, end bodyweight; ADG, average daily gain; ADFI, average daily feed intake; F/G, feed to gain ratio; L/M, lactulose/mannitol; CON, basal diet; ZnO, basal diet supplemented with ZnO (2,000 mg Zn/kg); C_ZnO, basal diet supplemented with C_ZnO (500 mg Zn/kg)*.

### C_ZnO Improved the Intestinal Mucosal Morphology

**Figure 1:**
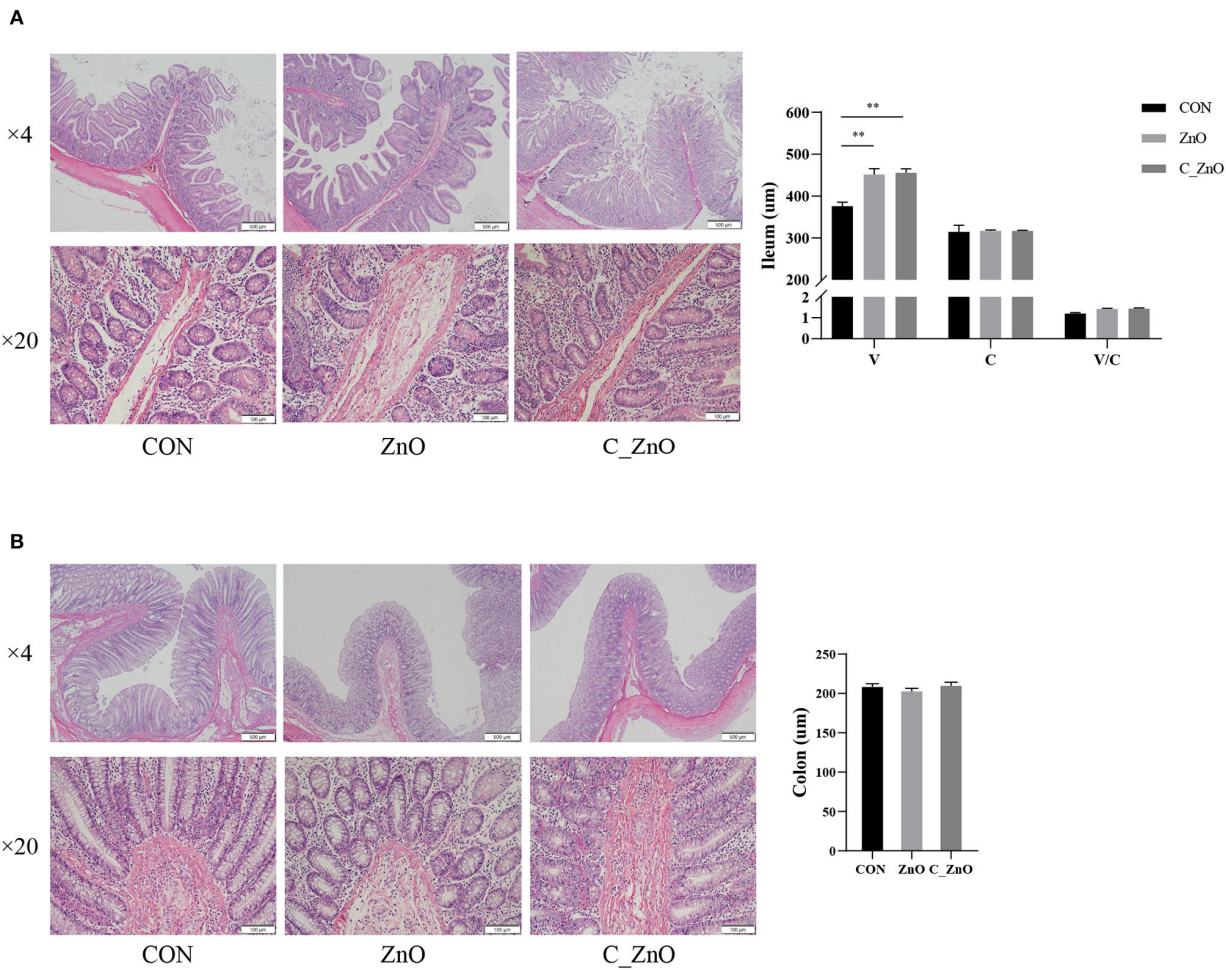
The effects of ZnO and coated ZnO (C_ZnO) on the intestinal mucosal morphology. **(A)** H&E staining of ileal tissue in piglets and the analysis of intestinal morphology. **(B)** H&E staining of colonic tissue in piglets and the analysis of intestinal morphology. Data are expressed as mean ± SEM, *n* = 6. ^*^P < 0.05, ^**^P < 0.01. CON, basal diet; ZnO, a basal diet supplemented with ZnO (2,000 mg Zn/kg); C_ZnO, a basal diet supplemented with C_ZnO (500 mg Zn/kg); V, villus height; C, crypt depth; V/C, the ratio of the villus height to crypt depth.

Coated ZnO significantly increased the villus height in the ileum (*P* < 0.01), while no difference was found in the ratio of villus height to crypt depth in the ileum and crypt depth both in foregut and hindgut (*P* > 0.05, Figure 1). And the improved effect of C_ZnO reached the same level of traditional ZnO in high dose.

### C_ZnO Strengthened the Intestinal Barrier Function

**Figure 2:**
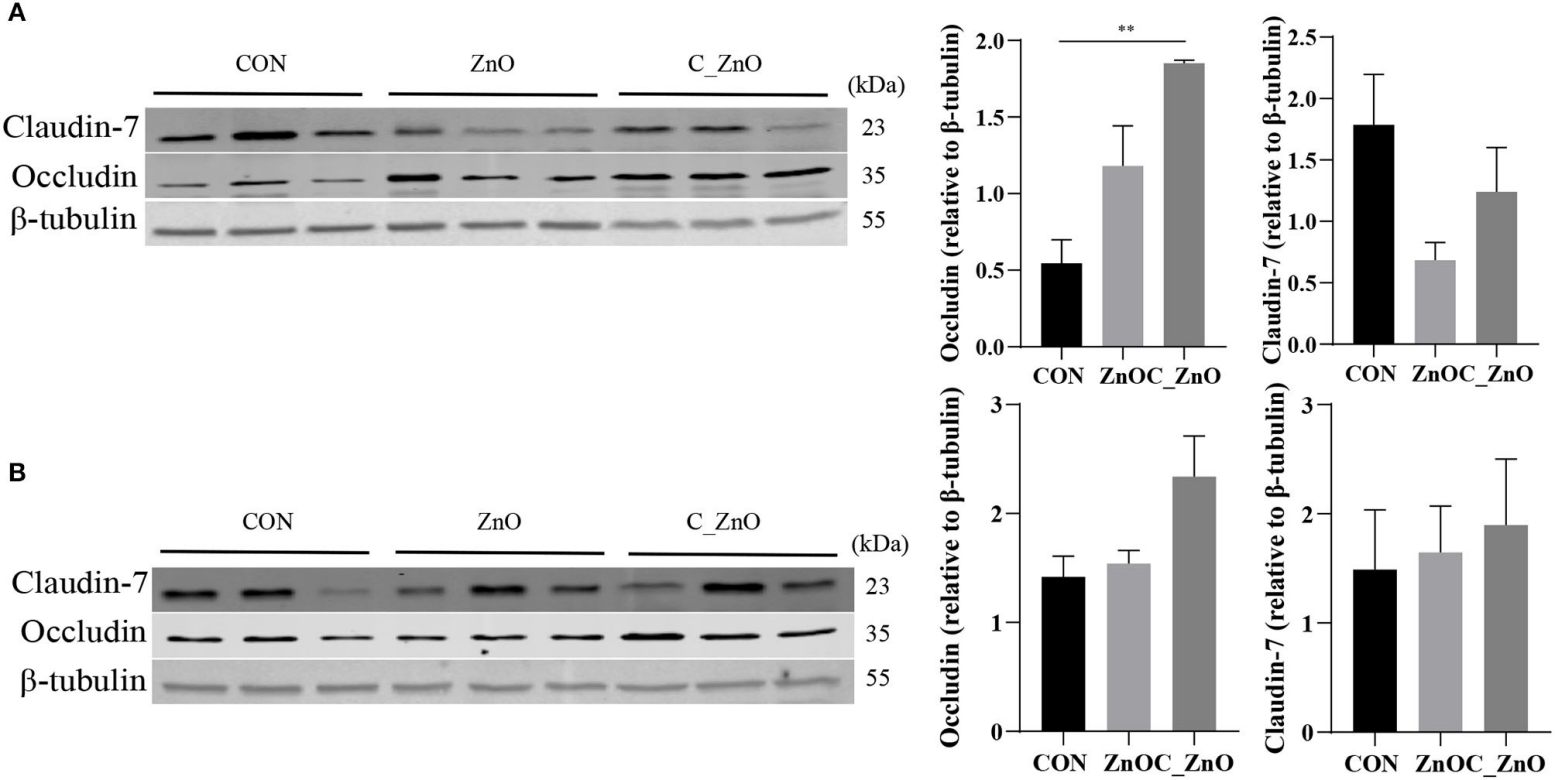
The effect of ZnO and C_ZnO on the expression of intestinal barrier proteins. **(A,B)** Western blotting results and analysis of Occludin and Claudin-7 in ileal **(A)** and colonic **(B)** tissues. Data are expressed as mean ± SEM, *n* = 3. ^*^P < 0.05, ^**^P < 0.01. CON, basal diet; ZnO, a basal diet supplemented with ZnO (2,000 mg Zn/kg); C_ZnO, a basal diet supplemented with C_ZnO (500 mg Zn/kg).

Coated ZnO significantly increased the expression of Occludin in the ileum, compared with the CON group (*P* < 0.01), and also led to an increased trend in the colon. However, the expression of Claudin-7 showed no difference among these groups (*P* > 0.05, Figure 2).

### C_ZnO Regulated the Composition and Diversity of the Bacterial Community

**Figure 3:**
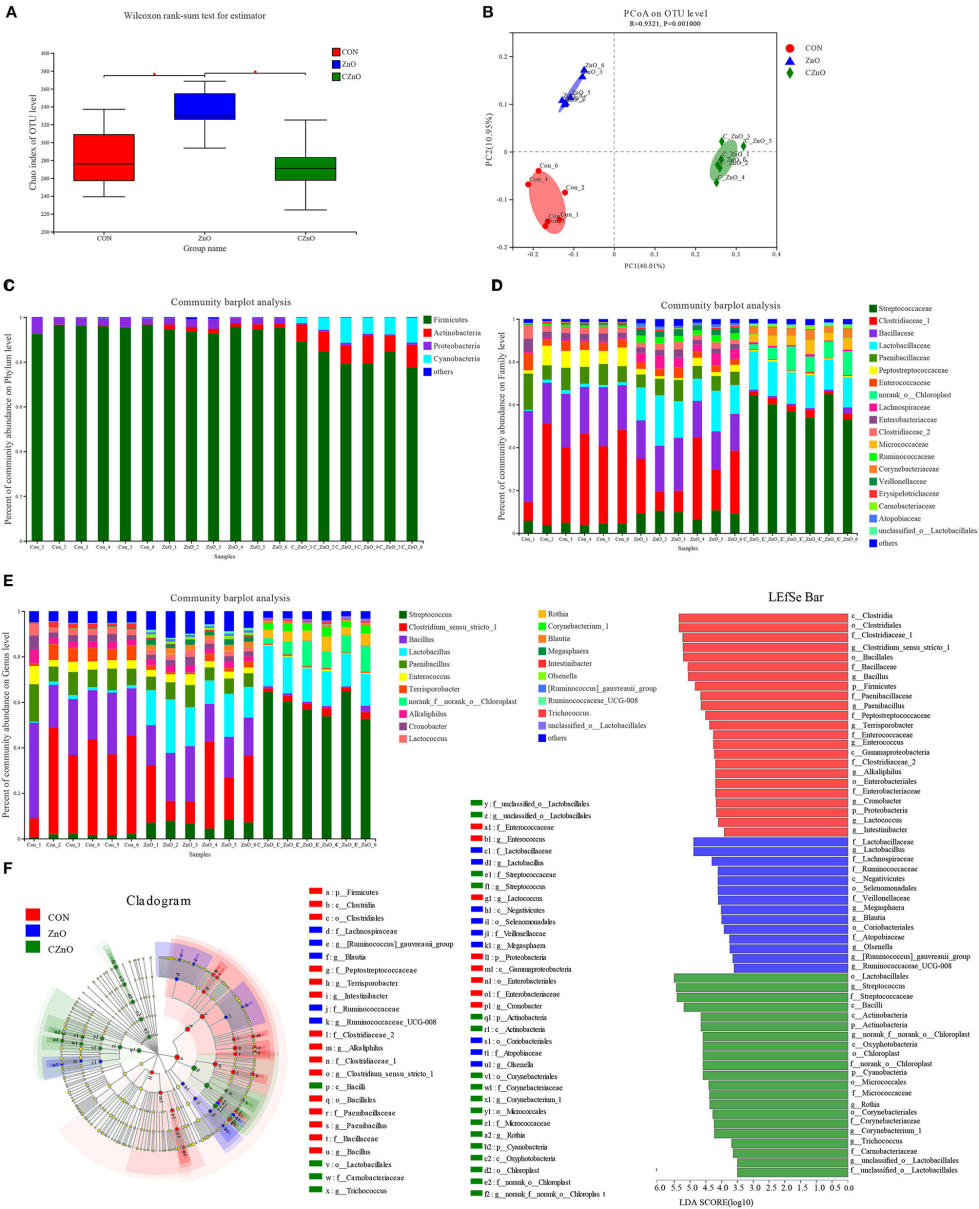
Effects of ZnO and C_ZnO on ileal microbial community of piglets. **(A)** Chao analysis of intestinal flora. **(B)** Principal coordinate analysis (PcoA) of intestinal microbiota. **(C–E)** Microbial community in the phylum **(C)**, family **(D)**, and genus **(E)** level. **(F)** LEfSe analysis and LDA score distribution histogram. CON, basal diet; ZnO, basal diet supplemented with ZnO (2,000 mg Zn/kg); C_ZnO, basal diet supplemented with C_ZnO (500 mg Zn/kg).

Ileum harbored increased bacterial richness with the ZnO treatment compared to the other groups which were reflected by the higher Chao index (*P* < 0.05, Figure 3A). Notable differences were found among the three groups as indicated by the principal coordinate analysis (PCoA) within the ileal lumen (Figure 3B). At the phylum level, C_ZnO treatment led to significant growth in Actinobacteria (*P* < 0.05) and Cyanobacteria (*P* < 0.05) and decrease in Proteobacteria (*P* < 0.05) in the foregut (Figure 3C). Down to the family level, compared to the other groups, the abundance of Streptococcaceae, norank_o__Chloroplast, Micrococcaceae, and Corynebacteriaceae (*P* < 0.05) were increased, accompanied by decline of Clostridiaceae_1, Bacillaceae, Paenibacillaceae, Enterococcaceae, Enterobacteriaceae, and Clostridiaceae_2 (*P* < 0.05) in the ileal lumen. Moreover, C_ZnO and ZnO treatments both induced a remarkable increase of Lactobacillaceae (*P* < 0.05, Figure 3D). In the genus level, C_ZnO had more *Streptococcus, Rothia*, and *Corynebacterium_1* (*P* < 0.05) and a lower abundance of *Clostridium_sensu_stricto_1, Bacillus, Paenibacillus, Enterococcus, Alkaliphilus*, and *Cronobacter* (*P* < 0.05) than CON and ZnO groups. The changes in genus level were consistent with the overall changes in the family level (Figure 3E). Linear discriminant analysis effect size (LEfSe) bar also showed specific enrichment of genus *Rothia* (linear discriminant analysis [LDA] score: 3.89) in C_ZnO group. And *Lactobacillus* and *Blautia* were significantly increased in ZnO group (LDA score: 4.89 and 4.01, respectively, Figure 3F; Supplementary Figure 1).

**Figure 4:**
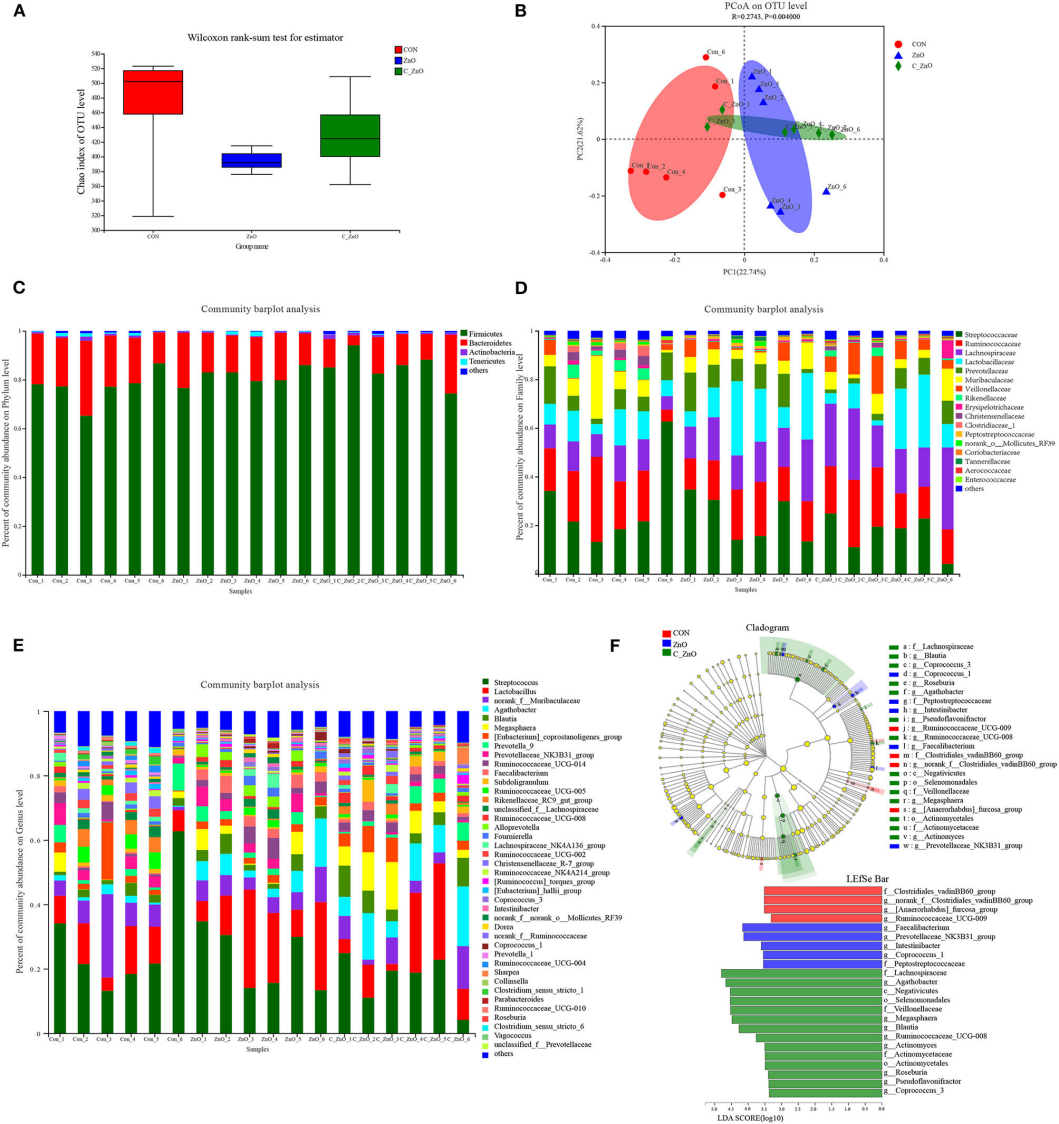
Effects of ZnO and C_ZnO on colonic microbial community of piglets. **(A)** Chao analysis of intestinal flora. **(B)** Principal coordinate analysis (PCoA) of intestinal microbiota. **(C,D)** Microbial community in the phylum **(C)**, family **(D)**, and genus **(E)** level. **(F)** LEfSe analysis and LDA score distribution histogram. CON, basal diet; ZnO, a basal diet supplemented with ZnO (2,000 mg Zn/kg); C_ZnO, a basal diet supplemented with C_ZnO (500 mg Zn/kg).

While in the colonic lumen, a relatively slight difference was found with an unchanged chao index though the PCoA plot showed separate clusters (Figures 4A,B). The microbial structures were kind of similar in the phylum level (Figure 4C), while the difference appeared in the lower level. The abundance of Streptococcaceae was dramatically increased in the CON group, which showed an opposite trend from the ileal lumen. Lachnospiraceae and Lactobacillaceae showed a slight increase in both ZnO and C_ZnO treatment, whereas the abundance of Clostridiaceae_1 was decreased. And the ZnO group gathered Prevotellaceae in the lumen compared to the C_ZnO group (Figure 4D). In the genus level, C_ZnO gathered more Agathobacter, *Roseburia*, and *Blautia* compared to ZnO. Furthermore, ZnO treatment raised *Faecalibacterium, Prevotellaceae_NK3B31_group, Intestinibacter*, and *Coprococcus_1* (Figures 4E,F). LEfSe analysis between two groups also reflected the results (Supplementary Figure 2).

### C_ZnO Increased the Concentration of Intestinal SCFAs

ZnO increased the concentration of acetate in the colon (*P* < 0.05) and propionate in both ileum (*P* < 0.05) and colon (*P* < 0.05), while C_ZnO significantly increased the concentration of acetate and propionate in ileum (*P* < 0.05) and colon (*P* < 0.05) compared with the other groups. Moreover, there was no difference in concentration of butyrate in ileum among the groups, whereas a decline of butyrate content was found in the colonic lumen of the ZnO and C_ZnO treatments (*P* < 0.05, Table 3).

**Table 3:** Effects of C_ZnO on intestinal SCFAs in the weaned piglets.

**Items**	**Treatment**		
	**CON**	**ZnO**	**C_ZnO**
**SCFAs of ileum, mg/g**			
Acetate	4.48 ± 0.26^b^	4.36 ± 0.21^b^	5.92 ± 0.34^a^
Propionate	2.25 ± 0.20^c^	2.54 ± 0.12^b^	3.81 ± 0.30^a^
Butyrate	0.18 ± 0.01	0.18 ± 0.02	0.24 ± 0.04
**SCFAs of colon, mg/g**			
Acetate	4.29 ± 0.06^c^	4.39 ± 0.03^b^	6.01 ± 0.07^a^
Propionate	2.37 ± 0.05^c^	2.61 ± 0.04^b^	3.79 ± 0.06^a^
Butyrate	1.73 ± 0.11^a^	1.04 ± 0.02^b^	1.06 ± 0.10^b^

*Data were shown as mean ± SEM (n = 6). Different superscript within a row means significantly different (P <0.05)*.

*SCFA, short chain fatty acid; CON, basal diet; ZnO, basal diet supplemented with ZnO (2,000 mg Zn/kg); C_ZnO, basal diet supplemented with C_ZnO (500 mg Zn/kg)*.

### *Streptococcus thermophilus* Possessed C_ZnO and ZnO Tolerance and Could Inhibit the Proliferation of *Bacillus cereus*

**Figure 5:**
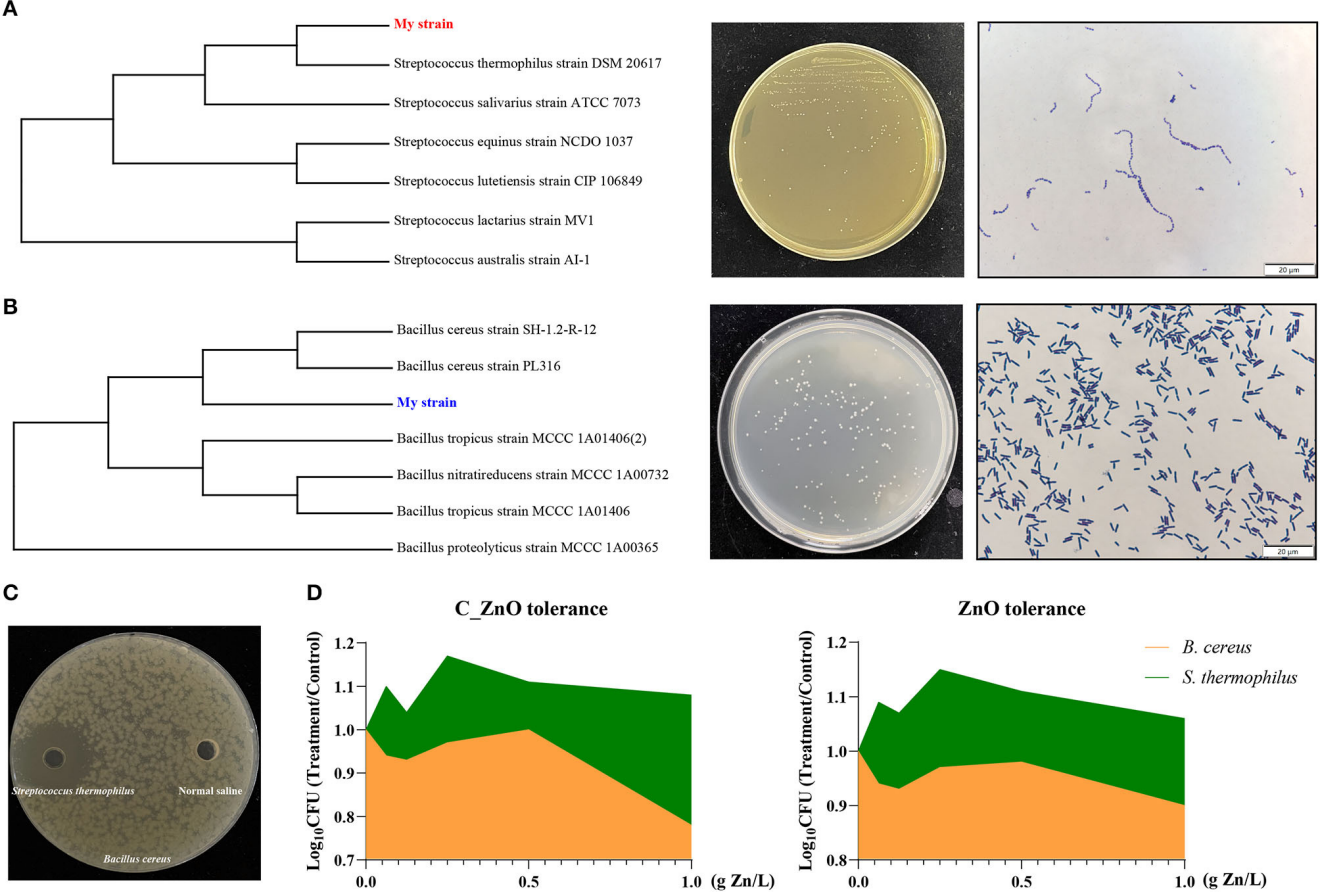
Bacterial isolation, characterization, and physiological identification. **(A)** Phylogenetic tree, colonial morphology, and Gram staining of *S. thermophilus*. **(B)** Phylogenetic tree, colonial morphology, and Gram staining of *B. cereus*. **(C)** Antibacterial activity of *S. thermophilus*. **(D)** Tolerance of C_ZnO and ZnO in *S. thermophilus* and *B. cereus*.

After isolation and identification, we obtained two strains from the ileal digesta of C_ZnO treated piglets. One shared the closest genetic relationship with *S. thermophilus*, gram-positive bacteria with smooth colony cultured on MRS (Figure 5A), while the other was detected as *B. cereus*, gram-positive bacteria with snowflake colony isolated from LB (Figure 5B). Except for the strong antibacterial capacity of *S. thermophilus* (Figure 5C), it was also promoted by not only C_ZnO but ZnO even in high concentrations of 1 g Zn/L, whereas the *B. cereus* was inhibited under the same condition (Figure 5D).

## Discussion

Coated ZnO reached a better effect than traditional ZnO on ADG and feed efficiency even in a lower dose, which coincided with the abundance of previous data (3, 18). And the statistical result of ADFI proved that neither ZnO nor C_ZnO affected palatability. What is more, the relieved diarrhea incidence resulting from ZnO and C_ZnO treatment was also in line with the notable effect of ZnO (19, 20).

Diarrhea was accompanied by ruined villi and a slow cell renewal rate, leading to disorders of nutrient absorption, eventually causing growth retardation in weaned piglets (21). In the present research, ZnO and C_ZnO both elevated the villus height in the foregut, which broadened the intestinal absorption surface area. However, there was no significant difference in crypt depth, which commonly reflected the rate of intestinal renewal and secretion function (22), and the index reflected absorption capacity, the ratio of villus height to crypt depth, was not affected either.

Changed intestinal permeability commonly contributed to the imbalanced fluid and electrolytes, which finally caused diarrhea (23). The lower L/M indicated that ZnO and C_ZnO decreased the small intestinal permeability. Intestinal permeability was partly affected by tight junctions (TJs), including claudins, Occludin, and junctional adhesion molecules, which formed at the apex of the basolateral membranes of epithelial cells to control the paracellular permeation (24). The paracellular pathway could divide into two different routes: one named “pore pathway” controlled by claudins, regulating the flux of small molecules and the other named “leak pathway” controlled by Occludin, regulating the transportation of large molecules (25, 26). TJs were also linked to intracellular located adaptor protein, such as zonula occludens, together which form the intestinal barrier (24). Our results found C_ZnO increased the expression of Occludin in the ileum, which led to low intestinal permeability and could enhance the resistance against the translocation of antigens (27).

Intestinal microbiota was crucial for host homeostasis (28). Our results found that C_ZnO mainly regulated the composition of ileal flora rather than colonic microbiota. Small intestinal communities were commonly overlooked because of the lower bacterial richness and diversity compared with the flora in the hindgut (29). However, many studies found that small intestinal microbiome was strongly associated with host diseases, such as inflammatory bowel disease and celiac disease, which broadened new sight to the exploration of disease pathogenesis based on the structure of small intestinal communities (29, 30). Compared to ZnO, C_ZnO increased more ileal Actinobacteria, one of the most frequent phyla in the gut, which was pivotal for health due to their function of immunity regulation and polysaccharide fermentation (31), which is related to the increased concentration of SCFAs in the lumen. Moreover, the reduced Proteobacteria in the C_ZnO group was mainly caused by decreased Enterobacteriaceae, commonly included enteropathogenic bacteria, such as *Escherichia coli, Shigella*, and *Cronobacter* (32, 33). Clostridiaceae was considered as commensal which comprised a series of metabolic capabilities, whereas, it also accommodated plenty of porcine enteric pathogens (34). The fluctuation of Clostridiaceae_1 in C_ZnO group was mainly due to *Clostridium_sensu_stricto_1*, which had been found to be related to neonatal necrotizing enterocolitis and colonic mucosal injuries (35, 36). Notably, C_ZnO and ZnO both significantly enriched lactate-producing microbes, *Streptococcus* and *Lactobacillus* in the ileal lumen. *Lactobacillus*, as a recognized probiotic, could promote intestinal health through different pathways, including pathogen inhibition, immunity regulation, and inflammation alleviation (37). The metabolite of lactic acid bacteria, lactate, also played a key role in the proliferation of intestinal stem cell, which protected the host from gut damage induced by stresses, such as chemotherapy and radiation (38). In accordance with the positive effect of C_ZnO on gut flora, the increased concentration of acetate and propionate also confirmed a more harmonious intestinal environment. *Streptococcus* had been found to be correlated with piglet body weight and commonly related to the healthy structure of microbiota (39, 40) in accordance with the improved growth performance in the group. Interestingly, our results also found *Streptococcus* was gathered in the colonic lumen in the CON group to some extent, rather than ileal lumen. Given that *Streptococcus* normally resided in the small intestine, the unusual microbiome transition indicated the disappearance of intestinal microbiome regionalization in the stress of weaning (41). Because of the drastic increase of *Streptococcus* in ileal lumen compared with other groups, we speculated that *Streptococcus* might be a mediator for the positive effect of C_ZnO. In addition, we also observed a remarkably opposite trend of *Bacillus* and *Streptococcus via* C_ZnO intervention. Thus, through isolation and identification, we obtained strains individually belonging to *S. thermophilus* and *B. cereus* from the ileal chyme of the C_ZnO treated piglets. In accordance with previous studies, *S. thermophilus* showed higher tolerance to C_ZnO and ZnO, which is partial due to their complex extracellular structure and mechanisms resisting toxicity of metal ions *via* biosorption and bioaccumulation (42). And this trait had drawn much attention to use as microbial nano-factory for metal particle production, which illustrated the booms of the genus in the foregut, whereas *B. cereus* was strongly inhibited in a similar environment. The intestine of livestock was a natural reservoir for foodborne bacteria, such as *B. cereus*, which not only caused widely contamination but triggered diarrhea and gastric ulcerations in the piglets, leading to the risk for growth retardation and even death (43, 44). Through the antibacterial experiment *in vitro*, we also found the strong inhibiting effect of *S. thermophilus* against *B. cereus*, which also verified the coexclusion effects. However, further research was needed to detect the function and interaction of the strains *in vivo*.

The fluctuation of colonic microorganisms seemed irregular, compared with the ileal microbiome. Meanwhile, combined with the result that the ileal microbiome was more similar between CON and ZnO groups, it reflected that C_ZnO mostly entered the small intestine as molecules through the protection of coating and its main cite for the function was the small intestine. Although, the colonic microbiome was relatively stable, some beneficial microbes still gathered in the treatment groups, such as the butyrate-producer, *Faecalibacterium* in the ZnO group and *Roseburia* and *Blautia* in the C_ZnO group, which was inconsistent with the decline of butyrate in the colon (45, 46). The result might be related to the cross-feeding effects of microbes. The increased abundance of these bacteria might be related to the booms of ileal *Lactobacillus* and *Streptococcus*, of which metabolite, lactate was their substate (47). Similar to the ileal environment, the content of acetate and propionate was also enriched in the colonic lumen. Studies had showed that acetate worked in the regulation of inflammation and in keeping from enteric infection (48). Except for building an acidic microenvironment which limited the growth of pathogens, acetate commonly served as a substrate for butyrate (47). Similar to acetate, propionate also showed the potential to resist inflammation and regulate colonic regulatory T cells (49). Considering postweaning diarrhea commonly had a relationship with inflammation and bacterial translocation, the enhanced SCFAs could partially illustrate the positive effect of C_ZnO in the piglets. Furthermore, unlike the ZnO treatment, C_ZnO could not gather Prevotellaceae, which functioned mostly on the fiber metabolism in the hindgut (50).

## Conclusion

Coated ZnO could improve growth performance and alleviate diarrhea in the weaned piglets, which was characterized by the improved intestinal barrier and intestinal morphology. Significantly, C_ZnO mainly adjusted the composition and structure of ileal microbiota, accompanied by increased concentration of SCFAs to exhibit its positive function. Moreover, compared to the high dose of ZnO, a low dose of C_ZnO did better in our research.

## Data Availability Statement

The datasets supporting the conclusions of this article are available in the NCBI Sequence Read Archive (SRA) repository under accession number PRJNA769193 (available on 1 March 2022).

## Ethics Statement

The animal study was reviewed and approved by the Animal Care and Use Ethics Committee of China Agricultural University (AW82011202-1-2, Beijing, China).

## Author Contributions

XM guided and designed the whole study. YS, NM, ZQ, and MH performed the experiment, including histological, chemical, and microbiological analysis and statistical analysis. The manuscript was mainly written by YS, and edited by XM. All the authors had read and approved the final version of this manuscript.

## Funding

This study was supported by the National Natural Science Foundation of China (31930106 and 31829004), the National Ten-thousand Talents Program of China (23070201), the Henan Province Public Benefit Research Foundation (201300111200-05), the 2115 Talent Development Program of China Agricultural University (1041-00109019), and the 111 Project (B16044).

## Conflict of Interest

The authors declare that the research was conducted in the absence of any commercial or financial relationships that could be construed as a potential conflict of interest.

## Publisher's Note

All claims expressed in this article are solely those of the authors and do not necessarily represent those of their affiliated organizations, or those of the publisher, the editors and the reviewers. Any product that may be evaluated in this article, or claim that may be made by its manufacturer, is not guaranteed or endorsed by the publisher.

## Supplementary Material

Supplementary Figure 1 LEfSe analysis of ileal microbiota between two groups. **(A)** LEfSe analysis between CON and ZnO group. **(B)** LEfSe analysis between CON and C_ZnO group. **(C)** LEfSe analysis between ZnO and C_ZnO group. CON, basal diet; ZnO, a basal diet supplemented with ZnO (2,000 mg Zn/kg); C_ZnO, a basal diet supplemented with C_ZnO (500 mg Zn/kg).

Supplementary Figure 2 LEfSe analysis of colonic microbiota between two groups. **(A)** LEfSe analysis between CON and ZnO group. **(B)** LEfSe analysis between CON and C_ZnO group. **(C)** LEfSe analysis between ZnO and C_ZnO group. CON, basal diet; ZnO, a basal diet supplemented with ZnO (2,000 mg Zn/kg); C_ZnO, basal diet supplemented with C_ZnO (500 mg Zn/kg).
