# Supplementary figures and images for: Coated Zinc Oxide Improves Growth Performance of Weaned Piglets via Gut Microbiota
Source: Front Nutr. 2022 Feb 25;9:819722. doi: 10.3389/fnut.2022.819722 (PMC8916703; doi:10.3389/fnut.2022.819722)

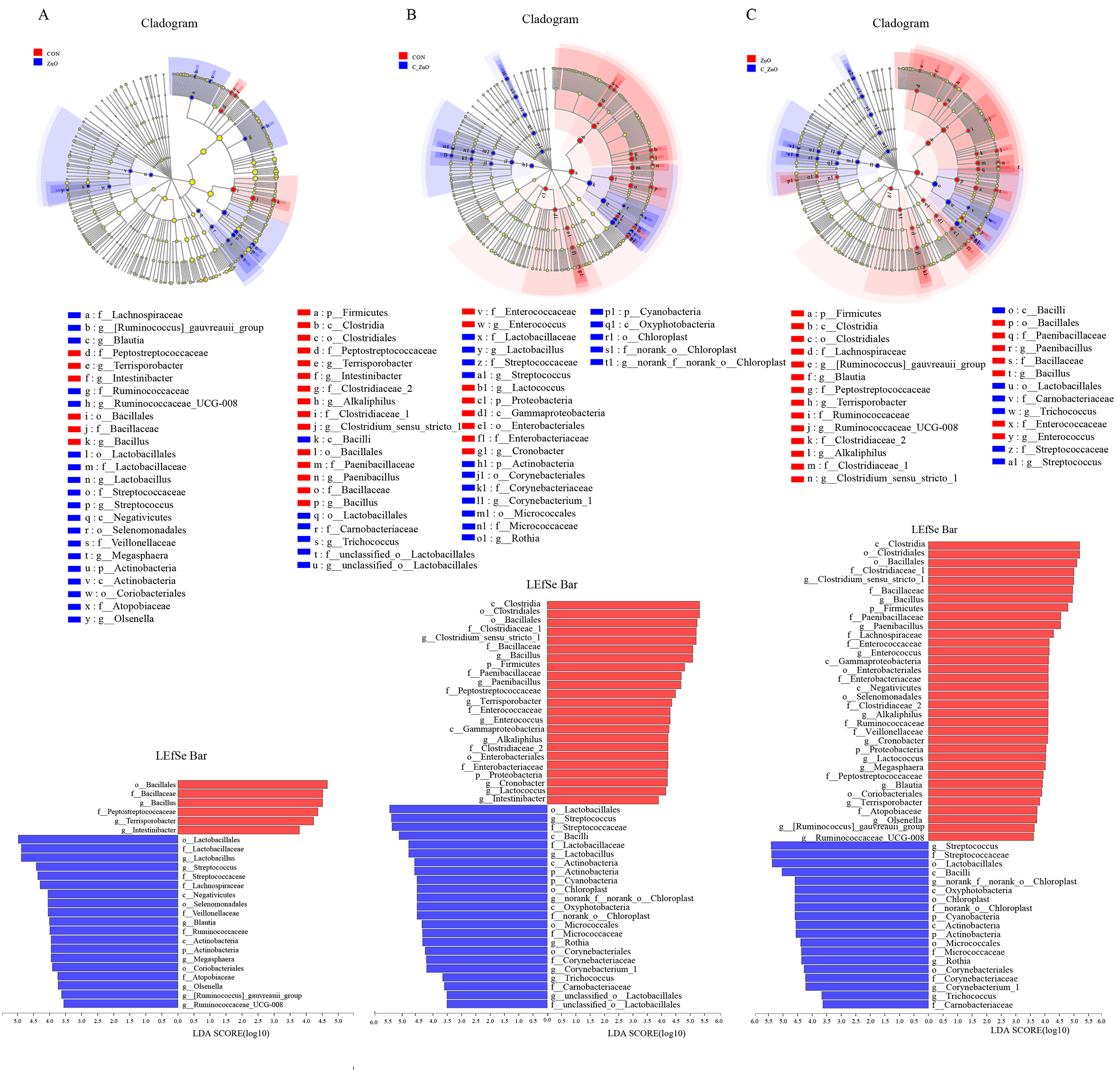

Supplement: Supplementary Figure 1 — LEfSe analysis of ileal microbiota between two groups. (A) LEfSe analysis between CON and ZnO group. (B) LEfSe analysis between CON and C_ZnO group. (C) LEfSe analysis between ZnO and C_ZnO group. CON, basal diet; ZnO, a basal diet supplemented with ZnO (2,000 mg Zn/kg); C_ZnO, a basal diet supplemented with C_ZnO (500 mg Zn/kg). [file Image_1.TIF]

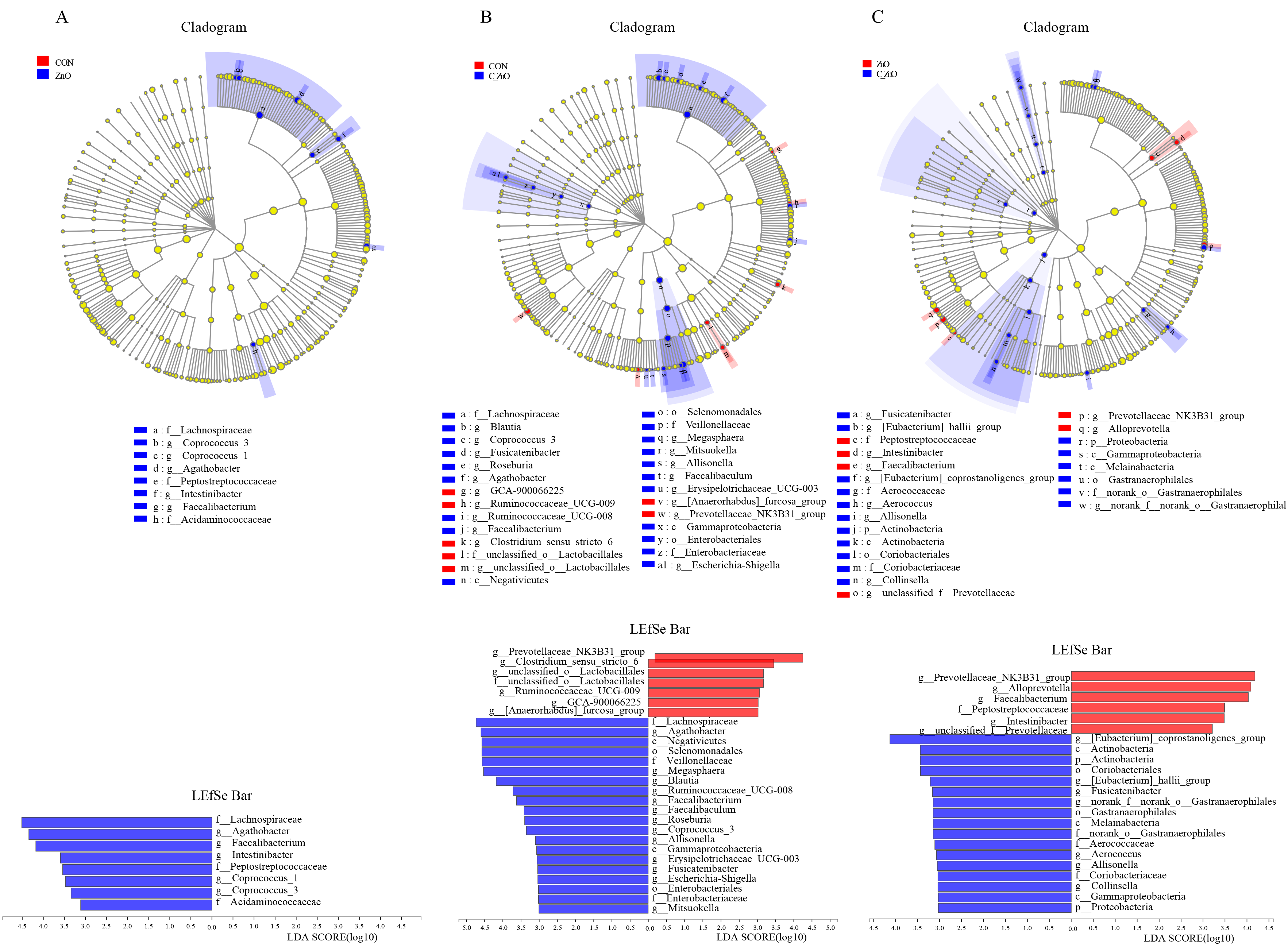

Supplement: Supplementary Figure 2 — LEfSe analysis of colonic microbiota between two groups. (A) LEfSe analysis between CON and ZnO group. (B) LEfSe analysis between CON and C_ZnO group. (C) LEfSe analysis between ZnO and C_ZnO group. CON, basal diet; ZnO, a basal diet supplemented with ZnO (2,000 mg Zn/kg); C_ZnO, basal diet supplemented with C_ZnO (500 mg Zn/kg). [file Image_2.TIF]
